# Biocontrol Effect and Antibacterial Mechanism of *Bacillus velezensis* TRMB57782 Against *Alternaria gaisen* Blotch in Korla Pears

**DOI:** 10.3390/biology14070793

**Published:** 2025-06-30

**Authors:** Chaowen Liu, Tiancai Wang, Yuxin Zhang, Hui Jiang, Xiaoxia Luo

**Affiliations:** 1 Key Laboratory of Protection and Utilization of Biological Resources in Tarim Basin of Xinjiang, Production Construction Corps, Alar 843300, China; 15593538060@163.com (C.L.); 14780650037@163.com (T.W.); 19936617672@163.com (Y.Z.); 2 School of Life Science and Technology, Tarim University, Alar 843300, China; 3 College of Chemistry and Chemical Engineering, Tarim University, Alar 843300, China

**Keywords:** biocontrol enzymes, secondary metabolites, volatile organic compounds, biocontrol mechanism, whole genome

## Abstract

Pear black spot disease seriously threatens the pear industry. Currently, its control mainly relies on chemical fungicides while biological control using antagonistic microorganisms represents a promising alternative approach. This study identified and characterized *Bacillus velezensis* TRMB57782 as a biocontrol strain through whole-genome sequencing. AntiSMASH analysis predicted the strain’s potential to produce secondary metabolites such as surfactin, difficidin, and bacilysin. In vitro experiments demonstrated that TRMB57782 inhibited the growth of *Alternaria gaisen*. In vivo experiments using excised branches and pear fruits at two different stages also showed significant control effects. A preliminary exploration of the metabolic substances of TRMB57782 was carried out. The strain can produce siderophores and three biocontrol enzymes. Crude extracts obtained by the hydrochloric acid precipitation and ammonium sulfate saturation precipitation of the bacterial liquid exhibited significant activity and volatile organic compounds showed biocontrol activity. Meanwhile, the effects of strain TRMB57782 on the hyphae of pathogenic fungi were studied, leading to hyphal atrophy and spore shrinkage. This paper provides an effective biocontrol strategy for fragrant pear black spot disease, reveals the antibacterial mechanism of *Bacillus velezensis* TRMB57782, and offers a new option for the green control of pear black spot disease.

## Simple Summary

Black spot disease in pears seriously threatens the pear industry. Currently, its control mainly relies on chemical pesticides, which not only disrupt the ecological balance but also lead to drug resistance in pathogenic bacteria. In this study, *Bacillus velezensis* TRMB57782 was isolated from the rhizosphere soil of *Tamarix chinensis* in the Taklamakan Desert, Xinjiang. It was found to effectively inhibit the growth of the pathogen causing pear black spot disease. The strain can produce various antibacterial substances including iron-binding compounds and biological enzymes. Its extracts and volatile components can cause the hyphae of the pathogen to shrink and spores to deform. The results provide an effective strategy and theoretical basis for the green control of pear black spot disease, helping to reduce the use of chemical pesticides and promote sustainable agricultural development.

## 1. Introduction

The Korla fragrant pear is a prominent fruit in Xinjiang [1,2]. It is highly susceptible to disease infection during cultivation, transportation, and storage, making this issue particularly prominent [3,4,5]. Fragrant Fire blight, black spot, and canker are the three major diseases affecting the fragrant pear industry. Among them, fragrant pear black spot is caused by *Alternaria gaisen* (*A. gaisen*). Upon infecting fruits, *A. gaisen* [5,6,7] can generate alternariol toxins, posing significant risks to human health. Pear black spot disease currently affects leaves, fruits, and new shoots, leading to rot during storage. This has become an urgent problem to be solved in the fragrant pear industry [8,9,10].

Currently, pesticide control remains the primary approach for managing pear black spot disease, with commonly used chemical agents including azoxystrobin, polyoxin, mancozeb, tebuconazole, and flusilazole [11,12,13]. The excessive use of chemical pesticides in the pear industry has created pressing issues. These agents disrupt the ecological balance, leading to enhanced drug resistance in pathogenic fungi and environmental pollution.

Biological control methods to reduce chemical pesticide use are key in national plant disease strategies. Biocontrol microorganisms are widely used for crop disease prevention due to their eco-friendly nature. Microbial pesticides effectively manage plant diseases and improve crop growth while reducing chemical dependency and promoting sustainable practices. The main physical prevention methods primarily focus on fruit bagging, sterilization with ultraviolet lamps [4], and tree removal, which are costly and ineffective. Biological control offers eco-friendly advantages, reduces pesticide resistance risks, and has become widely used in crop disease management. It represents a key approach for green plant disease prevention [14].

*Bacillus* species are common biocontrol bacteria [15] widely used in plant disease control due to stress resistance [16]. They form stress-tolerant spores and inhibit fungal growth via resource competition and antibacterial peptides, acting as eco-friendly agents without residues [17]. Antagonistic strains like *Bacillus cereus* BT8 [18] reduce Phytophthora capsici severity in cocoa while *Bacillus subtilis* TE3 [19] controls Bipolaris sorokiniana-induced wheat spot disease.

In addition to the above strains, many other *Bacillus* strains also show great biocontrol potential. Many *Bacillus* strains show strong biocontrol potential. Commercial Bacillus-based biopesticides like QST713 [20], GB03 [21], and DB9011 [22] from Agra Quest (Davis, CA, USA), Microbio Ltd. (Plano, TX, USA), and AHC Ltd. (Tokyo, Japan). are widely used. *Bacillus velezensis* (*B. velezensis*) demonstrates broad applications, with products such as Nufarm’s MBI600 (Ottawa, ON, Canada) [23] controlling fungal/bacterial/nematode diseases and Liaoning Tumugi’s TMQ-KSL-1 (Shenyang, China) [24] degrading Meloidogyne incognita eggs through serine protease 78.5% efficacy.

The current research on the biological control of pear Alternaria blotch using *B. velezensis* remains limited. While some *Bacillus* strains like *P. polymyxa* JE53 [25] and *B. tequilensis* 2–2a [26] show antifungal effects against pear pathogens, this study isolated *B. velezensis* TRMB57782 from Xinjiang *Tamarix chinensis* rhizosphere soil. Through morphological characterization, 16S rDNA analysis, and whole-genome sequencing, the strain was identified and its metabolic potential assessed. Fermentation experiments and antimicrobial tests demonstrated TRMB57782’s efficacy in controlling Korla pear Alternaria blotch with the preliminary exploration of its antibacterial mechanisms. These findings offer new biocontrol resources and support the further development of strain TRMB57782.

## 2. Materials and Methods

### 2.1. Microbiological Material

The TRMB57782 strain (JBNIIT000000000) was isolated from *Tamarix* rhizosphere soil in Taklamakan Desert. The pathogen of pear black spot disease, *A. gaisen* CGMCC3.7807, has been preserved in the laboratory of our research group and was purchased from the China General Microbiological Culture Collection Center (CGMCC).

### 2.2. Strain TRMB57782 Genetic Classification Status Determination

#### 2.2.1. Methods for Identification of Strain TRMB57782

The taxonomic classification of strain TRMB57782 was established through morphological examination, physiological and biochemical profiling, molecular examination, and molecular analysis. The strain was cultivated on LB plates using the streaking technique at a consistent temperature of 37 °C for 24 h, enabling the observation of morphological traits including colony color and shape. The strain TRMB57782 was collected by centrifugation, dried and gold-coated, and then observed using a scanning electron microscope [27]. Biochemical tests using API test strips (API-NE, API-50CHB and API-ZYM) [28] and reagents from bioMérieux (Marcy-l’Étoile, France) were employed to determine both oxidase activity as well as carbon and nitrogen source utilization for physiological and biochemical characterization. Following DNA extraction using the method of CTAB [29], sequencing was conducted by Shanghai Sangon Biotech Co., Ltd. (Shanghai, China). The 16S rDNA sequence was compared in the Ezbiocloud database [30] and 25 highly similar sequences were obtained. A phylogenetic tree was constructed using the maximum likelihood (ML) [31] method in MEGA X software (Version: 10.2).

#### 2.2.2. Methods for Comparative Genomic Analysis of Strain TRMB57782

Strain TRMB57782 was cultured in LB liquid medium at 37 °C and 180 r/min for 24 h. After cultivation, the bacterial solution was centrifuged at 4 °C and 12,000 r/min to collect the bacterial cells. The whole genome of TRMB57782 was sequenced by Nanjing Personal Biotechnology Co., Ltd. (Nanjing, China) using the Illumina NovaSeq 1.4 sequencing platform. The gene circle map was visualized using the online platform Proksee (https://proksee.ca/, accessed on 1 April 2025) [32].

A total of 19 strains with the highest similarity to TRMB57782 were selected and their complete genome reference sequences were downloaded from NCBI (National Center for Biotechnology Information, https://www.ncbi.nlm.nih.gov, accessed on 2 April 2025). These sequences, together with the genome of TRMB57782, were uploaded to the IPGA v1.09 [33] online platform (accessed on 2 April 2025) for comparative genomic analysis. The built-in analytical software of the platform was used with default parameters for pan-genome analysis, calculation of average nucleotide identity (ANI), and construction of a whole-genome phylogenetic tree based on core genes.

#### 2.2.3. Analysis of Antibacterial Potential of Strain TRMB57782 Based on Genomics

The antiSMASH [34] platform (https://antismash.secondarymetabolites.org, accessed on 4 April 2025) was utilized to conduct a systematic identification of secondary metabolite biosynthetic gene clusters in the genome of TRMB57782. Additionally, based on the CAZy database [35] (Carbohydrate-Active Enzymes Database, accessed on 5 April 2025), the HMMER 3.3.2 tool was employed to perform a scan for genes encoding biocontrol-related enzymes across the entire genome.

### 2.3. Strain TRMB57782 Biological Activity Determination

#### 2.3.1. Antimicrobial Effect Test of Fermentation Broths from Different Media on Pathogenic Fungi *A. gaisen*

The primary medium for generating high-yield active components [36] from strain TRMB57782 was screened and prepared according to Table S1. The test strain was inoculated and incubated at constant temperature of 37 °C and 180 rpm for a 4-hour culture period. When cultured on PDA (Potato Dextrose Agar) medium for 5–7 days, *A. gaisen* colonies typically exhibit gray–black to dark green colors with velvety surfaces. The fresh pathogen *A. gaisen* was evenly spread on a plate using the spreading method. Subsequently, six holes with a 7 mm diameter each were created on the pathogen-inoculated medium through the punching method. Each hole was then filled with 200 μL of one of six fermentation liquids using a kinds of pipette gun. The control group consisted of LB medium, with five replicate groups established. The diameter of the inhibition zone was measured.

#### 2.3.2. Evaluation of the Biocontrol Effect of Strain TRMB57782

(1) Determination of the Preventive and Therapeutic Effects on Detached Branches

Strain TRMB57782 was fermented in screening medium. The broth was adjusted to OD600 0.8–1.0 with sterile water (≈1 × 10^9^ CFU/mL test solution). Five days prior, pathogenic fungi *A. gaisen* were cultured in PDA media. Clear water served as negative control and 1:5000 diluted flusilazole as positive control as per instructions.

Healthy 25 cm shoots from Korla fragrant pear orchard were sterilized with 75% alcohol, rinsed thrice with sterile water, air-dried with paraffin-sealed ends. After creating 7 mm xylem-exposed wounds at shoot midpoints, TRMB57782 fermentation broth was applied. Following 3-hour air-drying, *A. gaisen* fungal plugs were inoculated. Shoots were incubated at 28 °C 60% humidity. Three groups (10 shoots each) had lesion lengths measured on days 7, 14, and 21 post inoculation to calculate disease index (1) and control efficacy (2).

Using the same preventive methods, Korla fragrant pear branches were pretreated and wounded, then inoculated with *A. gaisen* fungal agar plugs. After 3 days in a climate chamber, branches were sprayed with TRMB57782 broth and incubated at 28 °C. Three groups of 10 branches each were used. Branch and lesion lengths were measured at 7, 14, and 21 days. Disease index and control efficacy were calculated.

Pear black spot severity on excised branches was graded—Grade 0: no spots; Grade 1: spots on ≤1/3 branch length; Grade 3: spots on 1/3–2/3 length; Grade 5: spots on ≥2/3 length.

(1) $$Diseaseindex\;(\%)=\frac{\sum \left(number\;of\;branches\;at\;each\;level\times grading\;index\;value\right)}{total\;number\;of\;measured\;branches\times highest\;level}\times 100\%$$

(2) $$Controlrate\;(\%)=\frac{black\;spot\;area\;of\;control\;group-black\;spot\;area\;of\;experimental\;group}{black\;spot\;area\;of\;control\;group}\times 100\%$$

(2) Determination of the Preventive and Therapeutic Effects on Detached Young Fruits

Determination of preventive effect: In May, uniform healthy Korla fragrant pears (mass diff. ≤ 2 g) at Tarim University were aseptically processed. Fruits were sprayed with TRMB57782 ferment broth, dried for 3 h, then injected with 0.5 mL pathogen broth (3 mm depth) using sterile syringe. Fruits were stored in boxes with sterilized moistened cotton. Lesion areas were measured at 1, 7 and 14 days post inoculation to calculate efficacy. There were 5 fruits per group, with 3 replicates.

Determination of therapeutic effect: In vitro fruit aseptic treatment matched preventive effect protocols. After pathogenic fungi inoculation, fruits were incubated at 28 °C for 3 days until symptom onset. TRMB57782 broth was thoroughly sprayed post symptom emergence, followed by rewrapping and continuous cultivation. Disease progression was monitored, with symptom areas recorded at 1, 7, and 14 days post inoculation to calculate control efficacy (3).

(3) $$Control\;efficacy(\%)=\frac{Area\;of\;disease\;spots\;in\;the\;control\;group-Area\;of\;disease\;spots\;in\;the\;treatment\;group}{Area\;of\;disease\;spots\;in\;the\;control\;group}\times 100\%$$

(3) Determination of the Preventive and Therapeutic Effects on Ripe Fruits

Korla fragrant pears were collected from the same location. Other treatments followed the method described in young fruits, with preventive and therapeutic effects on ripe fruits being assessed.

### 2.4. Mechanisms of Strain TRMB57782 Against Pear Black Spot Pathogen

#### 2.4.1. Determination of Antibacterial Activity of Antagonistic Substance Crude Extract of Strain TRMB57782

After culturing strain TRMB57782 in LB medium for 48 h (OD_600_ = 0.8–1.0), crude extracts were obtained through four methods, each with control settings to validate specificity: HCl precipitation [37], 150 mL sterile fermentation filtrate adjusted to pH 2.0 with 12 mol/L HCl, incubated at 4 °C for 12 h, centrifuged at 12,000 r/min for 20 min, with precipitate dissolved in sterile water and neutralized to pH 7.0, with control using equal pH blank solution; ammonium sulfate precipitation [38], saturated ammonium sulfate added to 150 mL filtrate, incubated at 4 °C for 12 h, centrifuged, with precipitate dissolved in sterile water, with control prepared by dissolving equal volume of saturated ammonium sulfate solution in sterile water; and organic solvent extraction [39], with methanol/ethyl acetate at 1:3 ratio for ultrasonic extraction over 4 h, concentrated by rotary evaporation to yield medium-polarity and low/non-polarity extracts, with control using pure solvents.

Antimicrobial activity was evaluated via the plate-hole method: *A. gaisen* was spread on PDA plates, 7 mm holes were punched, and 200 μL of filtered extracts (0.22 μm sterile membrane) from the four methods were added. After 4-day incubation at 28 °C, inhibition zone diameters were measured in triplicate.

#### 2.4.2. Spatiotemporal Inhibitory Effects of VOCs on the Mycelial Expansion of Pear Black Spot Pathogen

The double-plate method [40] detected VOCs from TRMB57782. Three pear black spot pathogen agar plugs were inoculated on PDA medium. TRMB57782 on LB medium faced the pathogen culture, sealed. Blank control used uninoculated LB medium. Three replicates per treatment incubated at 28 °C. Pathogen colony diameters were measured at 3, 5, and 7 d for inhibition rate calculation.

#### 2.4.3. Qualitative Detection of Extracellular Enzyme and Siderophore Activity

TRMB57782 was inoculated on siderophore, protease, amylase, and cellulase medium plates [41,42]. After 3-day incubation at 28 °C, transparent zones were examined. Five replicates per treatment were conducted.

#### 2.4.4. Effect of Strain TRMB57782 on the Morphology of Pear Black—Spot Pathogen Hyphae

*A. gaisen* was inoculated on PDA medium using TRMB57782 strain cakes, with blank controls lacking the strain. Three replicates per group were incubated at 28 °C for 7 days. Hyphal samples from normal and antibacterial-affected areas were fixed overnight, dehydrated through ethanol gradients, dried, gold-coated, and analyzed via scanning electron microscopy to observe hyphal morphology changes.

### 2.5. Data Processing and Analysis

Experimental data underwent statistical analysis using Excel (Microsoft, Redmond, WA, USA) and SPSS 24.0 (IBM, Armonk, NY, USA) software at a significance level of *p* < 0.05. Significance difference analysis was conducted using Duncan’s new multiple range method, and GraphPad Prism 8.0 was employed for graphical representation.

## 3. Results

### 3.1. Determination of the Genetic Taxonomic Status of Strain TRMB57782

#### 3.1.1. Analysis of the Identification of Strain TRMB57782

**Figure 1:**
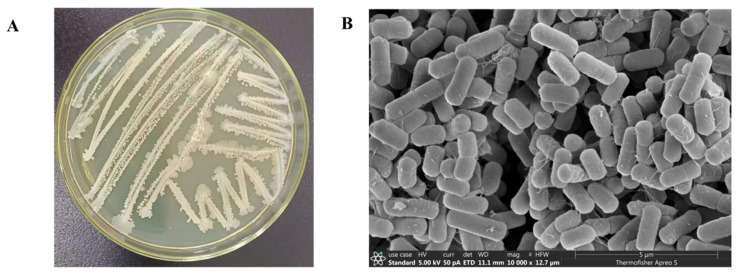
Morphological observation of strain TRMB57782. Note: (**A**) the growth of the colonies of strain TRM57782 after being streaked and inoculated for 24 h; (**B**) the morphology of bacterium TRM57782 under the electron microscope.

**Morphological Observation**: Strain TRMB57782 exhibited a milky white colony with a hint of gray on the LB plate, as depicted in Figure 1A. The colony was uniformly circular, possessing a waxy texture, smooth, and slightly convex, and measured between 1.0 to 1.5 mm in diameter. The texture was viscous. As illustrated in Figure 1B, magnified 1000× under a scanning electron microscope, the bacilli appeared rod-shaped, densely packed, and relatively uniform in size, and exhibited distinct surface textures.

**Physiological and Biochemical Indicators**: Significant variations in physiological and biochemical characteristics exist among strain TRMB57782, *B. velezensis* CR-502^T^, *B. subtilis* NCIB 3610^T^, and *B. siamensis* KCTC 13613^T^. Strain TRMB57782 differs from the comparative strains in its utilization of D-xylose, D-maltose, D-melezitose, and nitrate reduction, as well as in the activities of lipase and α-galactosidase (Table 1). It exhibits distinct behavior compared to *B. subtilis* NCIB 3610^T^ regarding lactose and citrate utilization, suggesting its potential classification as a novel species. The unique enzyme activities it possesses hold promise for applications in biodegradation and industrial enzyme production. Nevertheless, its incapacity to reduce nitrate may constrain its suitability in nitrogen cycle habitats.

**Figure 2:**
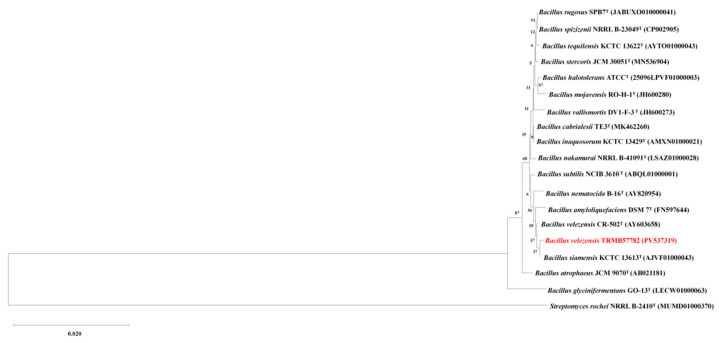
Phylogenetic tree of strain TRMB57782 constructed based on 16S rDNA sequence.

**Molecular Biological Identification**: Based on the 16S rDNA sequence, a phylogenetic tree was constructed using the maximum likelihood method (Figure 2), showing a close clustering of strain TRMB57782 and *B. velezensis* CR-502^T^ on the same branch with a high similarity of 99.71%.

In conclusion, through the comprehensive integration of colony morphology observations on the plate, the assessment of physiological and biochemical indicators, and the outcomes of molecular biological identification, strain TRMB57782 was conclusively recognized as *B. velezensis*.

#### 3.1.2. Comparative Genomic Analysis of Strain TRMB57782

**Figure 3:**
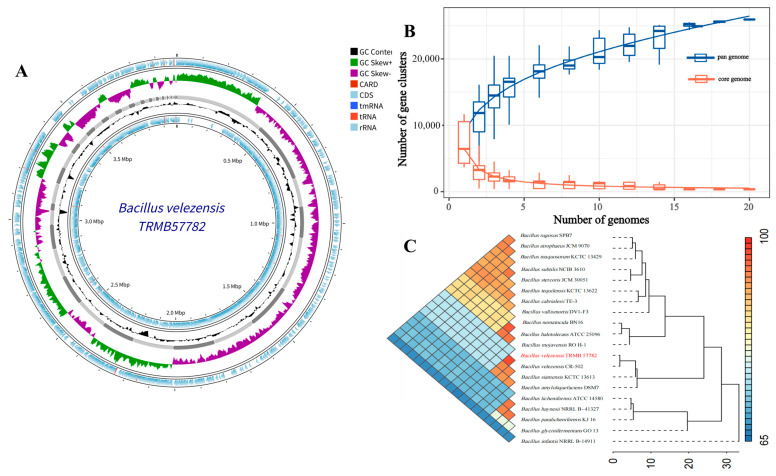
Genomic characteristics and comparative genomic analysis of *B. velezensis* TRMB57782. (**A**) The genome circle map illustrates *B. velezensis* TRMB57782. The innermost ring displays genomic coordinate markers, with GC Content (black) illustrating the distribution of GC content across the genome, and GC Skew+ (green) and GC Skew− (purple) indicating the skewed distribution of guanine (G) and cytosine (C) on the two DNA strands. The middle rings utilize various colors to represent gene elements: CARD (brown, antibiotic resistance genes), CDS (light blue, protein-coding genes), tmRNA (blue, transfer–messenger RNA), tRNA (orange, transfer RNA), and rRNA (light gray, ribosomal RNA). (**B**) Trends in the quantity of gene clusters for the pan genome (blue) and core genome (red) as the quantity of examined genomes rose. (**C**) Genome similarity heat map and clustering tree for 20 *Bacillus* strains, demonstrating genetic distances and evolutionary clustering relationships through average nucleotide identity (ANI) analysis.

Whole-genome sequencing of strain TRMB57782 revealed that the genome of *B. velezensis* TRMB57782 spans 3,984,114 bp, with a GC content of 46.48%. As shown in Figure 3A, it consists of 54 contigs, encompassing 3883 coding sequences (CDS), six ribosomal RNAs (rRNA), 59 transfer RNAs (tRNA), one transfer–messenger RNA (tmRNA), and eight drug-resistance genes, indicating a comprehensive array of gene expression and environment adaptation elements. The manuscript asserts that the complete genome sequence of strain TRMB57782 (genome assembly ASM5010661v1) is available in the NCBI database under BioProject PRJNA1252362.

The strain exhibited a significantly open pan-genome with rich genetic diversity and notable environmental adaptation potential. It comprised 34 core genes, 236 soft core genes, 10,493 shell genes, and 16,802 cloud genes, totaling 27,565 genes. The core genome curve demonstrated stability, while the pan-genome curve displayed a consistent upward trend as more genomes were analyzed, indicating ongoing gene discovery and robust environmental adaptation throughout evolution.

The ANI value for TRMB57782 and *B. velezensis* CR-502^T^ was 98.20%, indicating a significant genetic relationship. Additionally, the ANI values with *Bacillus siamensis* KCTC 13613^T^ and *Bacillus amyloliquefaciens* DSM7^T^ were 94.05% and 93.95%, respectively, demonstrating a notable level of genomic similarity. The whole-genome phylogenetic tree, constructed using core genes, demonstrated that TRMB57782 and *B. velezensis* CR-502^T^ formed a distinct evolutionary branch with a close genetic relationship. However, they exhibited significant divergence in their evolutionary trajectory compared to the other two strains, suggesting the accumulation of unique genetic variations over long-term evolution, resulting in distinct genomic characteristics from closely related strains.

#### 3.1.3. Elucidating the Antibacterial Potential of Strain TRMB57782 Through Genomic Analysis

AntiSMASH analysis was utilized to investigate the metabolic capabilities of *B. velezensis* TRMB57782, revealing 23 biosynthetic gene clusters (BGCs) across 12 categories in its genome (Table S2). Among these, BGCs with high similarity (≥77%) comprised bacillaene (100%), fengycin (80%), plantazolicin (91%), bacilysin (100%), bacinapeptin (100%), bacillibactin (100%), bacillothiazol A-N (100%), and macrolactin H-E (77%). The surfactin BGCs in Regions 8.1, 9.2, and 37.1 exhibited similarities of 47%, 47%, and 8%, respectively. Core gene cluster analysis indicated that these regions likely contain complete surfactin BGCs. Similarly, Regions 11.1 (46%) and 28.1 (26%) were identified as potential hosts for complete difficidin and its homolog BGCs. Predictions also suggested the presence of complete fengycin BGCs in Regions 1.2 (80%), 34.1 (20%), and 36.1 (13%). These compounds, including surfactin (lipopeptide), difficidin (polyketide), bacillaene (polyketide), fengycin (lipopeptide), bacilysin (peptide), bacillibactin (siderophore), and bacillothiazol A-N (thiazole-containing NRP), are known for their reported antifungal activities, particularly against *A. gaisen* [46,47,48].

**Figure 4:**
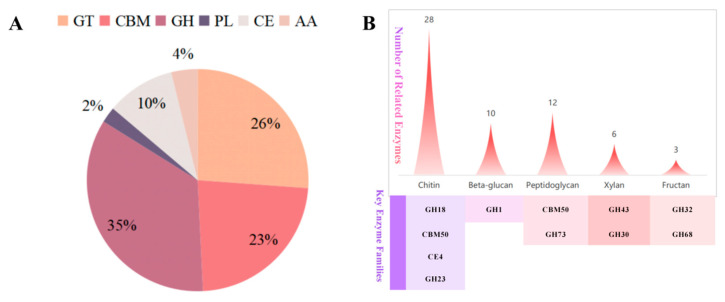
Statistical chart of the distribution of different enzyme families and the number of substrate-related enzymes. (**A**) Distribution statistics of different enzyme families. (**B**) Statistics of substrate-related enzyme quantities, key enzyme families, and their functional annotations.

*B. velezensis* TRMB57782’s genome contains 130 enzyme-related gene clusters with a focused CAZymes system effective against fungal pathogens. The GH family (34.62%) works with CBM and CE families to target fungal cell wall components (Figure 4A). Chitin-related enzymes dominate substrate-specific activity (Figure 4B), aligning with chitin’s importance in *A. gaisen* cell walls. GH18 chitinases and CBM50 modules (16.9%) form complementary networks, with CBM50 showing strong chitin-binding and GH18 exhibiting chitin degradation. GH1, GH5, and GH16 families target β-glucans by breaking glycosidic bonds. GH18 and CBM50 synergize through direct chitin degradation and substrate-binding enhancement, indicating antifungal potential against *A. gaisen*.

*B. velezensis* TRMB57782 demonstrates significant antibacterial activity against *A. gaisen* through the combined action of secondary metabolites and its CAZymes system.

### 3.2. Strain TRMB57782 Activity Determination

#### 3.2.1. Antibacterial Effect Test of Different Culture Media Fermentation Broth on Pathogenic Fungi *A. gaisen*

**Figure 5:**
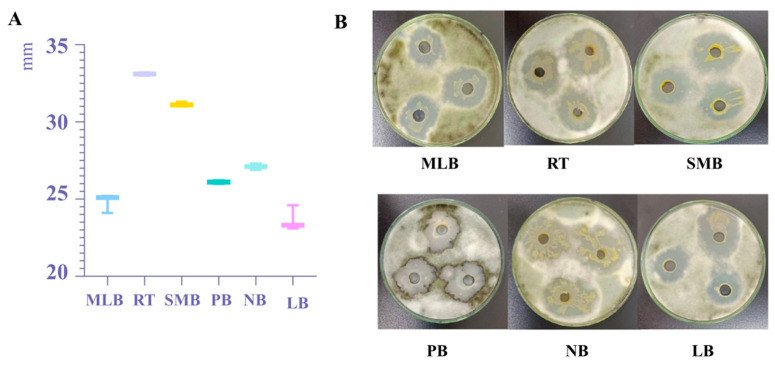
Screening of the basal medium of *Bacillus* TRMB57782 based on antibacterial activity components. (**A**) The diameter of the inhibition zone (unit: mm) of strain TRMB57782 in six different media was measured. (**B**) The growth of strain TRMB57782 and the morphology of the inhibition zone on six different media are presented. Each Petri dish had three holes, which facilitated three experimental repetitions.

Using the antibacterial activity of strain TRMB57782 as a reference, the diameter of the inhibition zone was assessed through the plate-hole punching technique to identify the medium with superior activity. The results demonstrated, as depicted in Figure 5, that the RT medium exhibited the highest efficacy, with an inhibition zone diameter of 3.31 ± 0.01 mm, representing a 39.66% increase compared to the LB medium. In addition, the diameter of the inhibition zone on the SBM medium was slightly lower at 3.11 ± 0.01 mm, indicating a 31.22% decrease in antagonistic activity compared to the RT medium. Other optimal media such as NB (2.71 ± 0.02 mm), PB (2.61 ± 0.01 mm), and MLB (2.48 ± 0.05 mm) experienced increases of 14.35%, 10.13%, and 4.64% respectively.

#### 3.2.2. Biocontrol Effect Determination

(1) Investigation into the preventive and curative impacts on detached branches

This study assessed TRMB7782 fermentation broth’s efficacy against pear black spot disease (Table 2). The results demonstrated significant disease control, with lower disease indices compared to sterile water and flusilazole controls. The broth maintained high effectiveness across most time points, confirming its preventive and therapeutic potential for infected branches.

At 7 days post treatment, the fermentation broth demonstrated a control efficacy of 80.67 ± 2.89%, significantly exceeding flusilazole’s 74.67 ± 1.53%. This indicates the superior therapeutic performance of the bacterial solution during early disease stages, effectively curbing disease progression. By day 14, while disease indices matched between bacterial solution and sterile water controls, the bacterial solution maintained higher control efficacy (88.33 ± 1.53%) compared to flusilazole (85.67 ± 5.78%). The trend continued through day 21 with bacterial solution efficacy remaining elevated at 86.33 ± 5.78% versus flusilazole’s 84.67 ± 2.52%, confirming consistent therapeutic stability against pear black spot disease. Throughout the 21-day prevention trial, strain TRMB57782’s fermentation broth maintained significantly lower disease indices than sterile water controls, with control efficacy stabilizing at elevated levels over time. These findings collectively demonstrate the bacterial solution’s robust preventive capabilities and sustained inhibitory effects on disease onset and development in affected pear branches.

(2) Investigation of Preventive and Curative Effects on Young Fruits throughout the Growth Phase

By measuring the prevention and control effect of the fermentation broth of strain TRMB57782 on pear black spot disease in young fruits during the growth period, the experimental results showed that the fermentation broth had a remarkable effect on preventing and treating pear black spot disease in young fruits (Table 3). From day 7 to 14, disease spot expansion slowed significantly, with control efficacy exceeding 90% compared to the water control, demonstrating strong protective effects during early fruit development. Compared to flusilazole, the bacterial solution showed comparable performance by day 14 (96.5% vs. the chemical’s 96.5%). In therapeutic applications, the bacterial solution achieved 78.89% efficacy on day 7 (vs. 82.60% for flusilazole), narrowing to 81.70% vs. 84.17% by day 14, indicating similar disease control capacity to chemical pesticides.

In conclusion, the fermentation broth of strain TRMB57782 showed positive effects on pear black spot disease in young fruits during the growth period, both in prevention and in treatment.

(3) Investigation of the Preventive and Curative Effects on Fruits throughout the Storage Duration

The study measured TRMB57782 strain fermentation broth’s efficacy against pear black spot during storage. The results demonstrated that it prevented and treated ripe pear black spot disease, with prevention outperforming treatment (Table 4).

During storage, the bacterial solution’s preventive effect on pear black spot disease increased from 40.19% on day 3 to 72.65% by day 14, showing delayed disease progression. Flusilazole outperformed significantly initially (96.07% vs. 40.19% on day 3), though the efficacy gap narrowed over time. In treatment, the bacterial solution maintained 27.43–60.51% control from days 3 to 14, demonstrating disease suppression capability but less effectiveness than its preventive action.

In conclusion, the fermentation broth of strain TRMB57782 has a better preventive effect than therapeutic effect in the prevention and treatment of black spot disease in ripe pears during the storage period. However, there is still a certain gap in effect compared with the chemical pesticide flusilazole.

### 3.3. Mechanisms of Strain TRMB57782 Antagonizing Black Spot Disease in Pears

#### Determination of Antibacterial Activity of Antagonistic Substance Crude Extract by Strain TRMB57782

**Figure 6:**
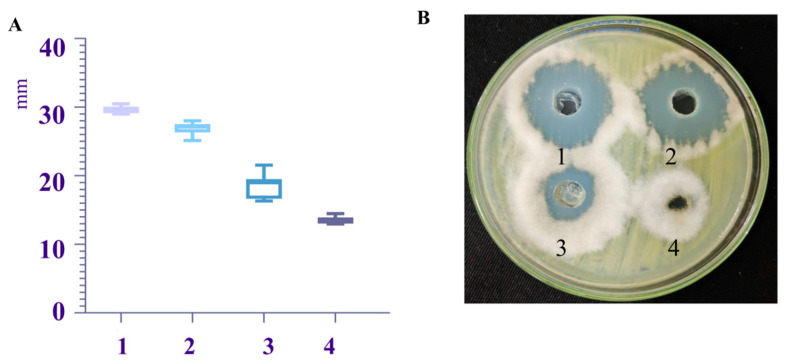
The antibacterial properties of crude extracts derived from the fermentation of *Bacillus* strain TRMB57782 using various extraction techniques. (**A**) Effect of crude antibacterial extract from strain TRMB57782 fermentation on pear black spot pathogen. (**B**) Antibacterial zone diameter statistics (mm) by method. Colored bars/error lines show inhibition zones from different extraction methods: 1: crude lipopeptide; 2: crude protein; 3: medium-polarity; 4: low/non-polar extract (*n* = 3).

The bacteriostatic substances in the fermentation filtrate of the biocontrol strain TRMB57782 were extracted using four different methods, and their activities against *A. gaisen* were assessed, as depicted in Figure 6. Crude extracts 1–3 showed significant inhibition zones of (33.20 ± 0.67) mm, (26.33 ± 0.81) mm, and (26.33 ± 0.81) mm, respectively. Acid precipitation extract 1 demonstrated the strongest activity, followed by ammonium sulfate precipitation extract 2, while ethyl acetate extract 4 showed no effect. The active components against *A. gaisen* appeared to be lipopeptides, proteins, and moderately polar compounds.

### 3.4. Spatiotemporal Inhibitory Effects of VOCs on the Mycelial Expansion of Pear Black Spot Pathogen

**Figure 7:**
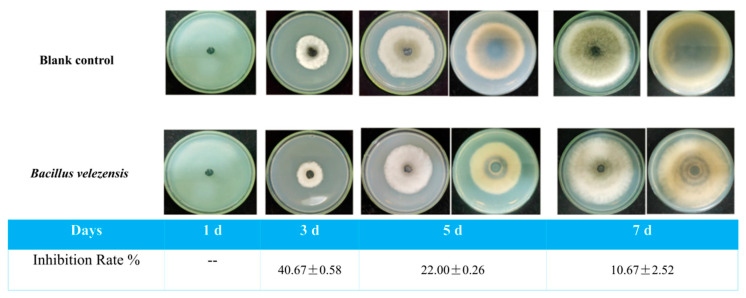
The imapact of VOCs produced by strain TRMB57782 on *A. gaisen* at various incubation periods. Study examined TRMB57782’s VOCs’ effect on *A. gaisen* growth across culture durations (1 d, 3 d, 5 d, 7 d). Upper row shows control group; lower row displays TRMB57782 inoculation impact. Controls observations were conducted at 5 d and 7 d intervals (*n* = 3).

The strain TRMB57782 was evaluated for volatile compounds using the double-plate method (Figure 7). VOCs from *B. velezensis* TRMB57782 inhibited *A. gaisen*, with rates decreasing from 40.67 ± 0.58% at 3 days to 10.67 ± 2.52% by 7 days, indicating weakening but persistent inhibition. Control colonies grew continuously while the experimental group showed smaller colonies with inhibition zones. These VOCs demonstrated potential as biocontrol fumigants.

### 3.5. Qualitative Detection of Extracellular Enzyme and Siderophore Activity

**Figure 8:**
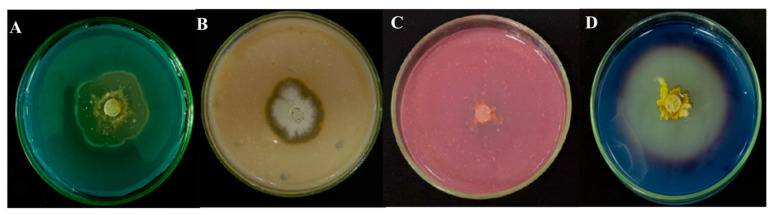
Production of extracellular enzymes by strain TRMB57782: (**A**) siderophores; (**B**) proteases; (**C**) cellulases; (**D**) amylases (*n* = 3).

The test results of strain TRMB57782, depicted in Figure 8, revealed its capacity to synthesize siderophores, proteases, cellulases, and amylases. Notably, amylase exhibited the highest activity, evidenced by a clear zone diameter of (6.59 ± 0.20) mm. The clear zone diameter for siderophore production was (4.41 ± 0.27) mm, indicating effective siderophore production. The protease activity showed relatively weak performance, with a clear zone diameter of (3.15 ± 0.055) mm, while the cellulase-producing ability was slightly weaker, with a clear zone diameter of (0.113 ± 0.058) mm.

### 3.6. Effect of Strain TRMB57782 on the Morphology of Pear Black Spot Pathogen Hyphae

**Figure 9:**
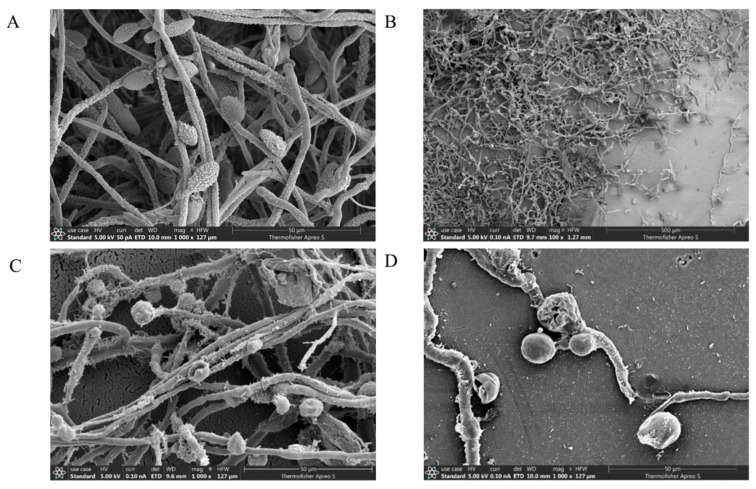
The impact of strain TRMB57782 on the mycelial morphology of *A. gaisen.* (**A**) The microscopic structure of *A. gaisen* under normal growth conditions at a magnification of 1000×. (**B**) The microscopic structure of *A. gaisen* at a magnification of 100× when inhibited by TRMB57782. (**C**) The microscopic structure of *A. gaisen* at a magnification of 100× (in the middle of the bacteriostatic zone) when inhibited by TRMB57782. (**D**) The microscopic structure of *A. gaisen* at a magnification of 1000× (at the edge of the bacteriostatic zone) when inhibited by TRMB57782.

Scanning electron microscopy revealed that TRMB57782 metabolites significantly affected *A. gaisen*’s mycelium. Normally, at 1000× (Figure 9A), *A. gaisen* showed smooth mycelium and inverted club-shaped spores with swollen bases and transverse septa. After TRMB57782 exposure (100×, Figure 9B), mycelial growth was sparse and stunted. At 1000× (Figure 9C,D), mycelia became uneven, atrophied, and twisted with reduced branching while spores decreased dramatically—many appeared shriveled and deformed. TRMB57782 inhibited both mycelial growth and normal morphological development.

## 4. Discussion

In recent years, global researchers have actively explored novel biocontrol resources from microorganisms in diverse environments to address environmental and health issues caused by the overuse of chemical pesticides [49]. The genus *Bacillus* has become a research hotspot in biocontrol due to its broad-spectrum antibacterial activity, stress resistance, and environmental friendliness. Among them, *B. velezensis* has gained significant attention since its establishment as an independent species in 2005, owing to its remarkable potential in agricultural disease control [43]. In terms of strain identification, precise microbial identification at the strain or species level still faces numerous challenges [50]. In this study, a *B. velezensis* strain TRMB57782 was isolated from the rhizosphere soil of *Tamarix* in the Taklamakan Desert, Xinjiang. Morphological observation showed that the strain formed milky-white colonies with regular edges on LB plates and appeared short-rod-shaped under an electron microscope, consistent with the typical characteristics of the genus *Bacillus* [51]. Physiological and biochemical analyses revealed that TRMB57782 utilized starch/mannitol as carbon sources and produced catalase, oxidase, and α-galactosidase, aligning with *B. velezensis* traits. The phylogenetic analysis of 16S rDNA showed 99.71% similarity to *B. velezensis* CR-502^T^ and taxonomy was further confirmed by ANI (average nucleotide identity) and genomic comparisons. Whole-genome sequencing identified functional genes/metabolic pathways, including antimicrobial lipopeptide clusters like Fengycin and Surfactin, supporting biocontrol research and strain development.

Black spot disease in fragrant pears, caused by *Alternaria* fungi, severely impacts yield and quality, leading to substantial losses for growers. Traditional chemical control provides short-term solutions but risks pathogen resistance and environmental pollution [52], making biocontrol a research focus. Strain TRMB57782 effectively inhibited *A. gaisen* in in vitro experiments and outperformed flusilazole in detached branch and fruit trials. Among six tested media, the RT medium significantly enhanced antibacterial activity, with inhibition zones of 3.31 mm—39.66% larger than those in the LB medium—suggesting that carbon/nitrogen ratios and Mg^2+^ may influence metabolite production by regulating lipopeptide synthesis genes [53,54].

The TRMB57782 fermentation broth showed superior efficacy against pear black spot disease compared to flusilazole in vitro (preventive 86.67%, therapeutic 86.33%). During young fruit development, its 96.27% prevention rate matched flusilazole’s 96.49% without residue concerns. However, efficacy decreased in stored mature fruits, potentially due to maturity-related metabolite permeability changes and ethylene-enhanced pathogen activity. This aligned with Wei’s finding that the cell-free filtrate of *B. velezensis* T3 [55] controlled black spot disease in cherry tomatoes but only delayed disease progression. TRMB57782 achieved > 90% control during growth phases but showed limitations after pathogen invasion in storage, indicating challenges in biocontrol following tissue penetration. Its environmental safety supports application in organic orchards, with early-stage use recommended, while formulation optimization could improve storage-phase performance.

The antimicrobial effects of *B. velezensis* originate from secondary metabolites encoded in its genome [56]. Whole-genome sequencing identified gene clusters in TRMB57782 for producing lipopeptides, polyketides, and non-ribosomal peptide synthetases, consistent with studies on *B. velezensis* T3 [55]. The acid-precipitated crude extract showed the highest antibacterial activity, primarily due to lipopeptides, while sulfate-precipitated proteins exhibited moderate efficacy. The strain also produced iron-chelating siderophores [57], proteases capable of degrading fungal cell walls, and amylases and cellulases—findings consistent with multiple studies showing that various *Bacillus* strains synthesize lipopeptides, a trait closely linked to their biocontrol potential. For example, *B. subtilis* Y17B [58] significantly inhibits *Alternaria* spp. by producing abundant secondary metabolites, particularly lipopeptides. Additionally, extracellular metabolites secreted by *B. amyloliquefaciens* LZN01 [59] in cell-free supernatants exhibit significant inhibitory effects against *Fusarium oxysporum*. VOCs of TRMB57782 maintained >10% hyphal growth suppression over seven days, potentially by interfering with fungal energy metabolism or cellular signaling mechanisms [60]. Studies have shown that benzothiazole and 2,4-dimethyl-6-tert-butylphenol produced by *B. velezensis* CT32 [61] strongly inhibit *Verticillium dahliae* and *F. oxysporum*. Electron microscopy confirmed that TRMB57782 metabolites caused hyphal shrinkage, spore deformation, and reduced branching in *A. gaisen*, indicating multi-mechanistic interference with fungal growth.

This paper has first reported the biocontrol potential of desert-habitat *B. velezensis* TRMB57782 against black spot disease in fragrant pears, revealed its multiple antibacterial mechanisms, and provided a scientific basis for green agricultural practices. Future research may focus on metabolomic analysis, field trials, and formulation development to promote the practical application of this strain.

## 5. Conclusions

A strain of *B. velezensis* TRMB57782 was isolated from Tamarix rhizosphere soil in Xinjiang’s Taklamakan Desert. Identified via morphological and biochemical features and similarity to *B. velezensis* CR-502^T^, it contains gene clusters for synthesizing lipopeptide antimicrobials like surfactin and fengycin. The strain’s optimized fermentation broth showed enhanced antibacterial activity, demonstrating 86.67% prevention on detached branches and >90% control efficacy against the Korla fragrant pear black spot pathogen during fruit development, outperforming flusilazole without pesticide residues. Its mechanisms include nutrient competition via siderophores, hyphal degradation through proteases, growth inhibition via volatile compounds, and fungal cell disruption by lipopeptides, with electron microscopy confirming hyphal deformation. While limited in post-harvest fruit protection, this study offered new biopesticide resources and theoretical foundations for desert microbial applications in agricultural disease control.

## Figures and Tables

**Table 1:** Physiological and biochemical indicators of the bacterial strains of TRMB57782.

Test Project	Result			
	TRMB57782	*B. velezensis* CR-502^T^ [43]	*B. subtilis* NCIB 3610^T^ [44]	*B. siamensis* KCTC 13613^T^ [45]
**Carbohydrate Acidogenesis**				
D-glucose	+	+	+	+
D-Xylose	-	+	-	+
L-Arabinose	+	+	+	+
D-Ribose	+	+	Ne	+
Lactose	+	+	-	+
Maltose	-	+	+	+
Sucrose	-	+	+	+
Glycogen	+	+	-	+
Inositol	+	+	+	+
Mannitol	+	+	+	+
Sorbitol	+	+	+	Ne
Salicin	+	+	+	+
D-Melezitose	+	Ne	Ne	-
D-Galactose	-	Ne	Ne	-
D-Fructose	+	+	+	Ne
**Nitrogen Source/Metabolism**				
Nitrate Reduction	+	+	+	+
Citrate Utilization	-	-	+	-
Urease	-	-	-	-
Esculin Hydrolysis	+	+	Ne	+
Gelatin Hydrolysis	+	+	+	+
**Enzyme Activity**				
Oxidase	+	+	+	-
Catalase	+	+	+	+
Starch Hydrolysis	+	+	+	+
Casein Hydrolysis	+	+	+	+

“+” indicates positive, “-” indicates negative, and “Ne” signifies the state of being undetermined.

**Table 2:** The curative and preventive effects of the fermentation broth of strain TRMB57782 on branches infected with *A. gaisen*.

Method	Treatment	7 d		14 d		21 d	
		Disease Index	Control Efficacy %	Disease Index	Control Efficacy %	Disease Index	Control Efficacy %
Prevention	Sterile water	20.00 ± 0.00 a	——	58.76 ± 6.11 a	——	98.67 ± 2.31 a	——
	Bacterial solution	8.67 ± 1.16 b	75.00 ± 3.27	10.67 ± 1.16 b	86.00 ± 0.82	12.67 ± 2.31 c	86.67 ± 0.47
	Flusilazole	6.00 ± 2.00 c	74.00 ± 0.82	16.67 ± 1.16 b	85.00 ± 0.00	19.33 ± 1.16 b	86.33 ± 0.47
Treatment	Sterile water	20.00 ± 0.00 a	——	55.33 ± 4.16 a	——	98.67 ± 2.31 a	——
	Bacterial solution	8.00 ± 2.00 b	80.67 ± 2.89	55.33 ± 4.16 a	88.33 ± 1.53	14.00 ± 4.00 b	86.33 ± 5.78
	Flusilazole	10.00 ± 2.00 b	74.67 ± 1.53	17.33 ± 1.16 b	85.67 ± 5.78	19.33 ± 1.16 b	84.67 ± 2.52

Data in the table are means ± standard deviations. Different lowercase letters after the data in the same column indicate significant differences at the *p* < 0.05 level by Duncan’s new complex polarity test (*n* = 3).

**Table 3:** The prevention and treatment effects of the fermentation broth of strain TRMB57782 on young fruits infected with pear black spot disease.

Method	Treatment	7 d		14 d	
		Lesion Area	Control Efficacy %	Lesion Area	Control Efficacy %
Prevention	Sterile water	13.11 ± 6.35 a	-	37.03 ± 8.02 a	-
	Bacterial solution	1.13 ± 0.04 b	91.38	13.82 ± 0.06 b	96.27
	Flusilazole	1.13 ± 0.09 b	98.52	13.00 ± 0.02 b	96.49
Treatment	Sterile water	125.83 ± 29.79 a	-	270.50 ± 8.78 a	-
	Bacterial solution	26.56 ± 7.70 b	78.89	49.50 ± 4.61 b	81.70
	Flusilazole	21.89 ± 1.53 b	82.60	42.83 ± 1.35 b	84.17

Data in the table are means ± standard deviations. Different lowercase letters after the data in the same column indicate significant differences at the *p* < 0.05 level by Duncan’s new complex polarity test (*n* = 3).

**Table 4:** Preventive and therapeutic impacts of the fermentation broth of strain TRMB57782 on fragrant pear infected with pear black spot during the storage period.

Method	Treatment	3 d		7 d		14 d	
		Disease Index Control Efficacy %		Disease Index	Control Efficacy %	Disease Index	Control Efficacy%
Prevention	Sterile water	105.43 ± 3.94 a	-	530.87 ± 131.78 a	-	1225.86 ± 422.05 a	-
	Bacterial solution	60.05 ± 8.17 b	40.19	183.94 ± 9.85 b	65.35	335.18 ± 17.03 b	72.65
	Flusilazole	4.14 ± 0.12 c	96.07	39.64 ± 10.15 b	92.53	200.51 ± 18.09 b	83.64
Treatment	Sterile water	85.67 ± 5.01 a	-	527.20 ± 26.98 a	-	1245.06 ± 68.56 a	-
	Bacterial solution	48.11 ± 4.09 b	43.84	382.57 ± 38.21 b	27.43	753.44 ± 24.30 b	60.51
	Flusilazole	37.07 ± 2.77 c	56.72	218.38 ± 31.84 c	58.58	428.20 ± 44.10 c	65.61

Data in the table are means ± standard deviations. Different lowercase letters after the data in the same column indicate significant differences at the *p* < 0.05 level by Duncan’s new complex polarity test (*n* = 3).

## Data Availability

The supporting data used in this study are available from the corresponding author upon request.

## Supplementary Materials

The following supporting information can be downloaded at: https://www.mdpi.com/article/10.3390/biology14070793/s1. Table S1. Formulas of six culture media used for the fermentation of strain TRMB57782; Table S2: Natural product synthesis gene cluster of strain TRMB57782.
