# Supplementary material for: Biocontrol Effect and Antibacterial Mechanism of Bacillus velezensis TRMB57782 Against Alternaria gaisen Blotch in Korla Pears
Source: Biology (Basel). 2025 Jun 30;14(7):793. doi: 10.3390/biology14070793 (PMC12292782; doi:10.3390/biology14070793)
Supplement: Supplementary file 1 [file biology-14-00793-s001.zip › biology-3680770-supplementary.pdf]

Table S1. Formulas of six culture media used for the fermentation of strain TRMB57782

Serial Number	Name	Formula (g/L)
1	MLB	Glucose 2.00, tryptone 7.00, yeast powder 2.00, NaCl 6.00, KCl 0.06, $MgCl_2 \cdot 6H_2O$ 0.50, with a pH value of 7.0. Sterilize at 141 ℃ for 20 minutes.
2	NB	Peptone 10.00, beef powder 3.00, sodium chloride 5.00, with a pH value of 7.0. Sterilize at 121 ℃ for 30 minutes.
3	PB	Peptone 12.00, yeast powder 1.00, glucose 15.00, $MgSO_4 \cdot 7H_2O$ 0.40, K_2HPO_4 0.05, with a pH of 7.0. Sterilize at 121 ℃ for 30 minutes.
4	SBM	Glucose 2.00, beef extract 4.00, yeast extract powder 4.00, $MgCl_2$ 8.00, KH_2PO_4 6.00, NaCl 0.01. Add 6 mL of 1 mol/L $CaCl_2$ and 6 mL of 1 mol/L $MnCl_2$. With a pH of 7.0. Sterilize at 115 ℃ for 20 minutes.
5	RT	Peptone 5.00, sucrose 10.00, yeast extract powder 1.00, beef extract 3.00, with a pH of 7.0. Sterilize at 115 ℃ for 20 minutes.
6	LB	Yeast extract powder 5.00, Peptone 10.00, NaCl 5.00, with a pH of 7.0. Sterilize at 121 ℃ for 30 minutes.

Table S2 Natural product synthesis gene cluster of strain TRMB57782

Region	Type	From	To	Most similar known cluster		Similarity
Region 1.1	transAT-PKS,T3PKS, NRPS	201,919	312,024	bacillaene	Polyketide+ NRP	100%
Region 1.2	NRPS,transAT-PKS, betalactone	369,059	456,849	fengycin	NRP	80%
Region 3.1	LAP,RRE-containing	170,541	193,718	plantazolicin	RiPP:LAP	91%
Region 5.1	other	177,710	219,128	bacilysin	Other	100%
Region 6.1	T3PKS	11,755	52,855			
Region 6.2	transAT-PKS-like	168,764	215,131	difficidin	Polyketide	53%
Region 7.1	terpene	54,666	75,406			
Region 7.2	PKS-like	158,764	200,008	butirosin A/butirosin B	Saccharide	7%
Region 8.1	NRPS	1	25,236	surfactin	NRP: Lipopeptide	47%
Region 9.1	lanthipeptide-class-iii	21,585	44,200	bacinapeptin	RiPP	100%
Region 9.2	NRPS	142,797	170,640	surfactin	NRP:Lipope ptide	47%
Region 11.1	transAT-PKS	123,680	157,780	difficidin	Polyketide	46%
Region 12.1	RiPP-like,NRP-metall ophore,NRPS	39,523	91,315	bacillibactin	NRP	100%
Region 14.1	NRPS	43,929	87,747	bacillothiazol A-N	NRP	100%
Region 21.1	transAT-PKS-like	1	46,043	macrolactin H/macrolactin B/macrolactin 1c/macrolactin E	Polyketide	77%

Region 22.1	terpene	8,031	29,914			
Region 23.1	NRPS	16,558	39,098	plipastatin	NRP	30%
Region 28.1	transAT-PKS-like	1	23,255	difficidin	Polyketide	26%
Region 34.1	NRPS	1	12,987	fengycin	NRP	20%
Region 36.1	NRPS	1	9,361	fengycin	NRP	13%
Region 37.1	NRPS	1	9,206	surfactin	NRP: Lipopeptide	8%
